# Stimulation of the tibial nerve: a protocol for a multicentred randomised controlled trial for urinary problems associated with Parkinson’s disease—STARTUP

**DOI:** 10.1136/bmjopen-2019-034887

**Published:** 2020-02-17

**Authors:** Doreen McClurg, Jalesh Panicker, Richard W Walker, AnneLouise Cunnington, Katherine H O Deane, Danielle Harari, Andrew Elders, Jo Booth, Suzanne Hagen, Helen Mason, Susan Stratton

**Affiliations:** 1 Nursing Midwifery and Allied Health Professions Research Unit, Glasgow Caledonian University, Glasgow, UK; 2 Department of Neurology, University College London, London, UK; 3 Department of Medicine, North Tyneside General Hospital, North Shields, UK; 4 Neurology Department, Glasgow Royal Infirmary, NHS Greater Glasgow and Clyde Clyde Division, Glasgow, UK; 5 School of Health Sciences, University of East Anglia, Norwich, UK; 6 Department of Ageing and Health, Kings College London, London, UK; 7 School of Health and Life Sciences, Glasgow Caledonian University, Glasgow, UK; 8 Yunus Centre, Glasgow Caledonian University, Glasgow, UK

**Keywords:** parkinson-s disease, urology, neuro-urology

## Abstract

**Introduction:**

Parkinson’s disease is the second most common chronic neurodegenerative condition with bladder dysfunction affecting up to 71%. Symptoms affect quality of life and include urgency, frequency, hesitancy, nocturia and incontinence. Addressing urinary dysfunction is one of the top 10 priority research areas identified by the James Lind Alliance and Parkinson’s UK.

**Objectives:**

Conduct a randomised controlled trial (RCT) targeting people with Parkinson’s disease (PwP) who have self-reported problematic lower urinary tract symptoms, investigating the effectiveness of transcutaneous tibial nerve stimulation (TTNS) compared with sham TTNS. Implement a standardised training approach and package for the correct application of TTNS. Conduct a cost-effectiveness analysis of TTNS compared with sham TTNS.

**Methods and analysis:**

An RCT of 6 weeks with twice weekly TTNS or sham TTNS. Participants will be recruited in 12 National Health Service neurology/movement disorder services, using a web-based randomisation system, and will be shown how to apply TTNS or sham TTNS. Participants will receive a weekly telephone call from the researchers during the intervention period. The trial has two coprimary outcome measures: International Consultation on Incontinence Questionnaire-Urinary Incontinence Short Form and the International Prostate Symptom Score. Secondary outcomes include a 3-day bladder diary, quality of life, acceptability and fidelity and health economic evaluation. Outcomes will be measured at 0, 6 and 12 weeks.

A sample size of 208 randomised in equal numbers to the two arms will provide 90% power to detect a clinically important difference of 2.52 points on the Internatioanl Consultation on Incontinence Questionnaire - Short Form (ICIQ-SF) and of 3 points in the International Prostate Symptom Score total score at 12 weeks at 5% significance level, based on an SD of 4.7 in each arm and 20% attrition at 6 weeks. Analysis will be by intention to treat and pre defined in a statistical analysis plan

**Ethics and dissemination:**

East of Scotland Research Ethics Service (EoSRES), 18/ES00042, obtained on 10 May 2018. The trial will allow us to determine effectiveness, safety, cost and acceptability of TTNS for bladder dysfunction in PWP. Results will be published in open access journals; lay reports will be posted to all participants and presented at conferences.

**Trial registration number:**

ISRCTN12437878; Pre-results.

**Strengths and limitations of this study**

- Fully powered randomised controlled study.
- Participants may become unblinded.
- Participants may want to switch arms.

## Introduction: background and rationale

### Lower urinary tract (LUT) symptoms in Parkinson’s disease

Parkinson’s disease is characterised predominantly by the motor complaints of bradykinesia, rigidity, rest tremor and gait disturbances. However, non-motor symptoms (NMS) are a common accompaniment^1^ with LUT symptoms reported in 38%–71% of people with Parkinson’s disease (PwP).^2^ These LUT symptoms are an important cause of morbidity and have a major impact on early institutionalisation and health-related costs.^3^ There also exists an association between nocturia and the risk for falls and hip fractures.^4–7^

Current treatments for LUT dysfunction in PwP are limited. It is likely that levodopa and other Parkinson’s medication affect bladder function; however, studies evaluating the effects of these medications on micturition have produced conflicting results.^8^ Currently, PwP may be offered advice on fluid intake and on behavioural treatment such as habit training or timed voiding with or without pelvic floor muscle training, but the evidence base supporting these techniques is limited.^9–13^ Antimuscarinic medication, competitively antagonise muscarinic acetylcholine receptors, resulting in detrusor relaxation, lower intravesical pressures and reduced storage symptoms. However, it has been found that PwP often discontinue their use due to side effects of nausea, dry mouth and constipation.^11^ More importantly, there is evidence to suggest a worsening in cognition and consciousness in susceptible neurological patients, and caution in prescribing is advised.^10 14^ Intravesical injections of botulinum toxin into the detrusor is sometimes considered in PwP and has been described in four small studies to date,^15–18^ which reported some improvement in symptoms.

For the reasons discussed, there is a need to explore options that are non-invasive and associated with minimal side effects.

### Patient involvement

A focus group of patients and carers of PwP and clinicians involved in treatment was held in London in 2017. During this meeting, the impact of bladder dysfunction on quality of life (QOL) was highlighted and also the lack of effective interventions. Transcutaneous tibial nerve stimulation (TTNS) was identified as a treatment option they would be willing to use, and a treatment regimen of twice weekly stimulation sessions for 6 weeks was preferred from the point of view of compliance, acceptability and practicability. We also have PwP on our trial steering committee, and they have advised us on the content of our participant facing literature and protocol.

### Tibial nerve stimulation (TNS): a form of neuromodulation

Neuromodulation has been used increasingly to treat urinary disorders through stimulation of the sacral, pudendal, tibial and genital nerves. The most often studied neuromodulation routes for overactive bladder (OAB) have been the sacral nerve roots and the tibial nerve (TN).^17^ Nerve fibres in the TN represent the L4-S3 spinal segments and therefore share a similar origin as the innervation to the bladder, rectum, anal sphincter and pelvic floor.^19^ The precise mechanism of action of neuromodulation is unclear, but it is thought to modulate the sacral plexus indirectly via the sensory, motor and autonomic fibres of the TN.^19^ Percutaneous TNS (PTNS) stimulation involves a qualified healthcare provider inserting a small needle just behind the medial malleolus to conduct the current from the small hand held stimulator. This is a ‘minimally’ invasive neuromodulation technique that is recommended by National Institute for Health and Care Excellence (NICE)^19^ for the treatment of OAB symptoms when conservative options have failed and is increasingly offered in hospital and urology services, requiring the patient to come into clinic for each treatment session (usually 12 weekly sessions). In comparison, much less is known about the effects of TTNS using the non-invasive transcutaneous route, which involves the delivery of the stimulation using surface electrodes that can be applied by the patient themselves and that can be used in the person’s home. TTNS is potentially more accessible and may enable PwP to self-manage and feel more in control of their care. It is also less costly than other stimulation modalities.

### Evidence

As stated above, TNS is a form of peripheral neuromodulation targeted towards symptom relief of OAB and urge urinary incontinence.^20^ The authors reviewed the literature relating to the use of TTNS or PTNS in the neurogenic population and identified one study with spinal cord patients in which the use of TTNS (n=50) was compared with solifenacin succinate (n=50). Both groups improved significantly in all bladder diary measurements, but there were fewer side effects with TTNS.^21^ Seth *et al* 22 compared two different regimens of TTNS (Gp1: 30 min daily; Gp2: 30 min weekly, for 12 weeks) between 24 patients with multiple sclerosis (PwMS) with OAB to 24 people with idiopathic OAB. Significant improvements in symptoms were reported for both groups. Another prospective study looked at TTNS in 70 PwMS with OAB over a 3-month period demonstrating efficacy of reduced urinary urgency and frequency as well as other secondary outcomes such as improved QOL and reduced burden.^23^ Several studies have reported TTNS use to be safe, acceptable and potentially beneficial in regards to LUT symptoms in PwMS in their own homes, older adults in care homes and stroke patients in their own homes,^22 24 25^ respectively. More recently, a study with 68 participants with overactive detrusor persisting after first-line or second-line treatments in a non-superiority trial reported no difference in efficacy between PTSN and TTNS.^26^

Studies specific to PwP and PTNS or TTNS are limited. Kabay *et al* 27 reported on a non-randomised study with 47 PwP and PTNS and found significant improvement in symptoms and urodynamic parameters. Only two small studies were identified with PwP using TTNS.^28 29^ Ohannessian *et al* 28 demonstrated that five out of six female PwP with OAB, who used TTNS for 6 weeks, considered TTNS to be effective. The study also implied that TTNS has potential efficacy of improving urodynamic and symptom scores in this population. Perissinotto *et al* 29 reported a study comparing TTNS and placebo stimulation in 13 PwP presenting with LUT symptoms. They demonstrated that TTNS holds promise as an option in the treatment of LUT symptoms in this population reducing urgency and nocturia and improving QOL.

The findings of this review have identified that TNS using the transcutaneous route (TTNS) is a feasible option for PwP, but efficacy has not been determined. A study is currently underway in France evaluating a TTNS regimen of 20 min daily stimulation for 90 days in a mixed group of patients with Parkinson’s disease and multiple system atrophy (MSA)^30^ Liaising with the Principle Investigator, recruitment into the study appears to be satisfactory. Considering that LUT dysfunction in Parkinson’s disease and MSA is distinctly dissimilar, we have chosen to study the effects of TTNS in Parkinson’s disease alone. Patient notes will be screened if the diagnosis is unclear. In addition, our consultation group of PwP and carers unanimously preferred a treatment regimen of twice weekly stimulation sessions for 6 weeks rather than daily sessions over 3 months from the point of compliance, acceptability and practicability. For this reason, we will investigate the most commonly used and evaluated treatment protocol for TNS involving 12×30 min stimulation sessions over 6 weeks.

It is hypothesised that the effectiveness and cost-effectiveness of a strategy of TTNS is superior to sham TTNS at 12 weeks.

### Objectives

The Stimulation of the tibail nerve for urinary incontinence in Parkinson's (STARTUP) trial will determine/undertake the following:

1. Conduct a randomised controlled trial (RCT) targeting PwP who have self-reported problematic LUT symptoms, investigating the effectiveness of TTNS compared with sham TTNS.
2. Implement a standardised training approach and package (already developed) for the correct application of TTNS.
3. Conduct a cost-effectiveness analysis of TTNS compared with sham TTNS.
4. Assess fidelity to the TTNS intervention and any research participation effects in the intervention and placebo stimulation groups.

### Trial design

The present study is a multicentred, parallel group, superiority, double-blind RCT comparing the effectiveness of an experimental strategy of twice weekly use of active TTNS for 6 weeks against a twice weekly use of sham TTNS for 6 weeks in PwP and LUT symptoms. Randomisation is at a 1:1 ratio for the two arms of the trial.

## Methods: participants, interventions and outcomes

Full administrative information is provided in table 1

**Table 1:** Administrative information

Trial registration with registry that adheres to WHO trial registration data set	ISRCTN12437878 https://doi.org/10.1186/ISRCTN12437878
Protocol version	Version 3 08/10/2019.
Funding	This study is supported by a grant from The Dunhill Medical Trust. Parkinson’s UK have provided additional support to cover NHS Support Costs.
Name and contact information for the trial sponsor	Yasmin Glover, RIE, Glasgow Caledonian University, Cowcaddens Road, Glasgow, G4 0BA, UK.
Role of sponsor	Glasgow Caledonian University is the sponsor and has the responsibility for overseeing the management and arranging the finance of the research. It must satisfy itself that the study meets the relevant standards and ensure that arrangements are put and kept in place for management, monitoring and reporting.
Corresponding author	Doreen.mcclurg@gcu.ac.uk
Reporting checklist	SPIRIT reporting guidelines were used to complete this protocol publication Chan A-W, Tetzlaff JM, Altman DG, Laupacis A, Gøtzsche PC, Krleža-Jerić K, Hróbjartsson A, Mann H, Dickersin K, Berlin J, Doré C, Parulekar W, Summerskill W, Groves T, Schulz K, Sox H, Rockhold FW, Rennie D, Moher D. SPIRIT 2013 Statement: Defining standard protocol items for clinical trials. Ann Intern Med. 2013;158(3):200–207.
Author conflicts of interest	No conflicts have been declared.

NHS, National Health Service.

### Study setting

Routine care setting for Parkinson’s patients in the UK.

### Eligibility criteria

#### Inclusion criteria

- Patients of 18 years of age and above (no upper age limit), with a diagnosis of Parkinson’s diesase(any stage) with self-reported problematic LUT symptoms.
- Capacity to consent/complete self-report outcome measures: ability to apply TTNS (or placebo) independently or has carer who can apply for duration
- Stable Parkinson’s medication for 3 months.
- Participants may be treatment naïve, failed or continuing treatment with antimuscarinic medication (group allocation will be minimised to account for these groups).
- Patients who are being treated, or have been treated, for benign prostatic hyperplasia or prostate cancer can be included at the discretion of the PI.
- Patients taking medications such as alpha-blocker medicines (prazosin, indoramin, tamsulosin, alfuzosin, doxazosin and terazosin), or 5-alpha reductase inhibitor medicines. Finasteride and dutasteride can be included. Patients taking approved treatment for prostate cancer, or example, apalutamide and enzalutamide, bicalutamide, Casodex, Zytiga, Lupron Depot, Xtandi and Zoladex can be included at the discretion of the PI.
- Patients who have had or are having treatment for cancer, for example, urological cancer can be included on an individual basis at the discretion of the PI.

#### Exclusion criteria

- Pacemaker or implanted electrical device, including deep brain stimulation.
- Unable to understand the instructions relating to the bladder diary and/or the use of the stimulator or does not have a relative willing to help.
- Ulceration or broken skin, in area of pad placement.
- History of peripheral vascular disease and epilepsy.
- Current urinary tract infection (if suspected by symptoms refer to General Practitioner and can recruit once cleared).
- Receipt of botox for bladder symptoms or TTNS within the last year.

### Who will take informed consent?

All participants will undergo a process of informed consent that will include the delivery of balanced written information concerning the need and overall benefit of the trial followed up by discussion with a local STARTUP researcher. This discussion will include a check of understanding concerning benefits and risks of participation and ensuring that participants accept that the treatment will be allocated at random regardless of any personal preference they may have.

Please see online supplementary material to review the consent form.

10.1136/bmjopen-2019-034887.supp1 Supplementary data

### Additional consent provisions for collection and use of participant data and biological specimens

Not applicable.

## The intervention

There is a risk of participant unblinding if the intervention or sham intervention is discussed in detail within this protocol; this will be published before trial recruitment is complete. The authors recognise the importance of the allocated intervention being accepted by the participant as the active intervention and have developed the sham intervention to be as realistic as possible yet delivering no intervention.

The treatment protocol developed by Amarenco *et al* 31 will be adopted for this trial.

All participants will use the stimulator for two 30 min sessions a week for 6 weeks. It is preferable to have 2–4 days between stimulation sessions.

A training protocol, including a manual for clinicians and instruction leaflets for participants, has been developed. Participants will be instructed to use the TTNS device twice weekly for 6 weeks with weekly telephone support by research staff.

### Criteria for discontinuing or modifying allocated interventions

There are no special criteria for discontinuing or modifying allocated interventions. Participants may choose to stop doing the active or sham TTNS themselves for any reason.

### Strategies to improve adherence to interventions

None beyond normal encouragement.

### Relevant concomitant care permitted or prohibited during the trial

No special provisions.

### Provisions for post-trial care

None beyond standard care within the National Health Service (NHS). Agreed to show how to set the parameters should the participants want to buy a unit.

### Outcomes

The outcome measures and other data to be collected are summarised in table 2.

**Table 2:** Summary of data collection

	Baseline (week 0)	Week 6–7 (on completion of intervention period	Week 12
ICIQ-UI SF	×	×	×
IPSS	×	×	×
3-day (24 hours) bladder frequency chart	×	×	×
Qualiveen	×	×	×
PDQ-8	×	×	×
Resource questionnaire		×	×
Change in medication	Weekly		×
Exit questionnaire		×	
Compliance downloaded		×	

ICIQ-UI SF, International Consultation on Incontinence Questionnaire-Urinary Incontinence Short Form; IPSS, International Prostate Symptom Score; PDQ-8, Parkinson’s Disease Questionnaire-8 items.

To decrease burden on participants (ie, only one visit), the baseline outcome data will be collected at the visit to clinic/home following completion of consent, randomisation and instruction on use of device.

#### Primary outcome measure

The trial has two coprimary outcome measures: International Consultation on Incontinence Questionnaire-Urinary Incontinence Short Form (ICIQ-UI SF)^32^ and the International Prostate Symptom Score (IPSS).^33^

These outcomes measure leakage (ICIQ-UI SF) and bladder over activity (IPSS) and were used to determine our sample size. They are validated for use in men and women.

The ICIQ-UI SF provides a brief and robust measure to assess the impact of symptoms of incontinence on QOL and outcome of treatment. It consists of four questions measuring frequency of UI, amount of leakage, overall impact of UI and when leakage occurs. It has grade A for validity, reliability and responsiveness to change established with rigour on one data set. The total score ranges from 0 to 21 with higher values indicating increased severity of symptoms.^32^

The IPSS is based on the answers to seven questions concerning urinary symptoms. The questions refer to the following urinary symptoms: (1) incomplete emptying; (2) frequency; (3) intermittency; (4) urgency; (5) weak stream; (6) straining; and (7) nocturia. Question 8 refers to the patient’s perceived QOL. Each question is assigned points from 0 to 5 indicating increasing severity of the particular symptom. The total score ranges from 0 to 35 (asymptomatic to very symptomatic). Although there are presently no standard recommendations for categorising the IPSS score, patients can be tentatively classified as follows: 0–7=mildly symptomatic; 8–19=moderately symptomatic; 20–35=severely symptomatic.^33^

#### Secondary outcome measures

- Qualiveen is an eight-item self-administered urinary QOL questionnaire validated in the neurogenic populations.^34^
- Parkinson’s Disease Questionnaire-8 (PDQ-8) is a validated eight-item self-administered questionnaire measuring Parkinson’s specific QOL.^35^
- A 72-hour bladder diary will be completed by participants during the week postrandomisation before commencing the use of the device at home (week 0), during week 7 and week 11 and will record frequency of micturition, leakage episodes and urgency. Participants will be advised to start the diary in the morning (first void of that day) following their clinic visit.
- Compliance: the stimulation unit will record how often and for how long the participant has used the unit during the 6 weeks of intervention, and data will be downloaded at clinic on receipt of the unit. The units are locked so that it is not possible for the participants to change settings or delete data.
- Resource use questionnaire completed by the participant at 6 and 12 weeks will monitor visits to the doctor/nurse/hospital, medications bought/prescribed or non-prescribed and purchases such as pads.
- Participant experience and protocol fidelity will be assessed at 6 weeks in a brief exit questionnaire.

Questionnaires and bladder diaries to be completed at 6 weeks will be given to the participant at visit 1, and those at 12 weeks will be posted to participants. All will be returned to the trial office in reply-paid envelopes. Should participants request, completion of the questionnaires may be done verbally by telephone.

### Participant timeline/pathway

The steps within the trial that the participants will take are summarised in box 1.

Box 1 **The participant pathway**

Participant’s identified at clinic by local PIs, Parkinson’s nurses or research nurses.

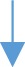

Participant information leaflet with expression of interest form attached given/sent to patient along with preaddressed envelope.

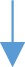

Posters also to be put up in relevant clinics to inform patients.

Potential participants opt in either by phoning the trial office or by returning the expression of interest form to them.

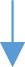

Staff at the trial office contact participant discuss further the implications of the study and complete screening log. If willing and eligible to be a participant, then the patient is allocated a participant study number and is given an appointment to attend the clinic or for the research nurse to visit them at home (confirmed by trial office with clinic)

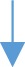

At the clinic/home, the participant will be consented, the clinical assessment form will be completed, randomisation will be completed (using the web-based system) and then the participant will be shown how to use the stimulation/placebo stimulation and provided with written instructions.

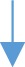

The participant is given a pack to take home with them containing the 3-day bladder frequency diary (with simple instructions) and the questionnaire booklet. The participant is asked to complete the bladder frequency diary during the next 3 days (starting with the first morning void) before starting to use the stimulation. The pack also contains the bladder diary and a questionnaire booklet to be completed at 6 weeks. Prepaid addressed envelopes will be available.

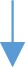

During the 3 days at home, the research office telephones the participant to support completion of their bladder diary. The participant either completes the questionnaires themselves or over the telephone. These diaries are then posted to the research office. Date when the participant will start using the intervention agreed and the times of the weekly follow-up telephone calls agreed.

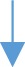

Trial office staff will telephone the participant weekly to see how things are going, ask about change in medications, adverse events and so on and will be available for any queries. Trial office will remind/help participant to complete the diary and questionnaires at the end of 6 weeks and will get the participant to unlock the compliance monitor before sending the unit back in the prepaid Jiffy bag.

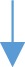

Trial office staff will post all the 12-week outcome questionnaires to the participant and telephone them to help with completion/complete over the phone depending on the participant’s preferences.

### Sample size calculation

A sample size of 208 participants randomised equally to the two arms will provide 90% power to detect a clinically important difference of 2.52 points^36^ on the primary outcome (ICIQ-UI-SF total score) at 6 weeks postrandomisation, using an independent samples t-test (two-sided significance level of 5%), based on an SD of 5 (equating to a standardised effect size of 0.5) and an attrition rate of 20% at 6 weeks. The SD of 5 is based on a recent systematic review of TTNS for OAB,^37^ where two trials reported ICIQ-UI-SF as an outcome measure, both with an SD of 4.3.^25 38^

For the coprimary outcome measure, the IPSS, the minimum clinically important difference is estimated to be 3 points^33 39^ and the systematic review of TTNS identified a single trial reporting IPSS, where the SD was 4.^40^ However, given the limited evidence available, we conservatively assume an SD of 6 points, which equates to a standardised effect size of 0.5. Therefore, a sample size of 208 will also provide 90% power to detect a clinically important difference in the IPSS.

## Recruitment

The research team at each study centre will be responsible for identifying potential participants and patients can self-refer in response to adverts by Parkinson’s UK and contact via patient research registers. Following the receipt of an expression of interest, a member of the research team will contact the patient by telephone to provide further information, assess eligibility and forward contact details to the relevant site.

### Assignment of interventions: allocation

#### Sequence generation

Participants who provide written informed consent will be randomised using a computer generated system to either TTNS intervention or non-active stimulation arm and will be minimised on two factors: (1) severity of urinary symptoms, as reported at study baseline by the IPSS in the Clinical Assessment Form, that is, mild, moderate or severe; and (2) status on antimuscarinic medication, that is, treatment naïve, failed or continuing such treatment.

#### Concealment mechanism

A web-based randomisation system will be used.

#### Implementation

Implementation will be by staff at the Centre for Healthcare Randomised Controlled trials Clinical Trials Unit (CTU) at Aberdeen.

### Assignment of interventions: blinding

#### Who will be blinded

Due to the nature of the intervention, the research team delivering the intervention will not be blinded to the treatment received; however, we have designed several different mechanisms by which participant allocation will be concealed. Participants will be blinded to group allocation. Outcome measures are primarily self-reported and submitted anonymously. Those involved in the data analyses and statistics will be blinded to the group allocation.

#### Procedure for unblinding if needed

Clinical staff are not blinded. At 6 weeks, the participant will be asked to which group they thought they had been allocated to assess the success of blinding. Data will be analysed by a statistician who is blinded to group allocation. At this stage, it they so wish, participants will be told to which group they had been assigned.

### Data collection and management

#### Plans for assessment and collection of outcomes

Data will be collected via participant-completed questionnaires at baseline and at 6 and 12 weeks. A 3-day bladder frequency diary will be completed prior to baseline, at 6 and 12 weeks. Completed outcome data are retuned by post. Relevant demographic and medical history information will be collected at baseline.

Patient resource use questionnaires will also be completed by participant at home at 6 and 12 weeks.

#### Plans to promote participant retention and complete follow-up

Nothing beyond normal encouragement to continue to use as prescribed. All participants will be offered help with setting the parameters of a stimulation unit should they purchase one.

### Data management

All trial participants are given a unique identifying trial number that will be used on all case report forms for that participant. Data will be entered into the secure trial database by the data coordinator based at the STARTUP central office at Glasgow Caledonian University.

#### Confidentiality

All investigators and study centre staff involved with this trial will comply with the requirements of the General Data Protection Regulations and the Data Protection Act 2018 in regards to the collection, storage, processing and disclosure of personal information.

Data collected during the course of the research is kept strictly confidential and accessed only by members of the trial team and may be looked at by individuals from the sponsor organisation or NHS sites where it is relevant to the participant taking part in this trial.

#### Plans for collection, laboratory evaluation and storage of biological specimens for genetic or molecular analysis in this trial/future use

None.

### Access to data

All data requests should be submitted to the corresponding author for consideration. Access to anonymised data may be granted following review.

## Analysis

All analyses will be clearly predefined, in agreed statistical and economic analysis plans, to avoid bias. Following data lock, analyses will be undertaken before unblinding of group allocation takes place. Analyses will be conducted according to the intention-to-treat principle, whereby all participants with follow-up data will be analysed according to their randomised allocation. A single main analysis will be performed at the end of the trial when all follow-up has been completed.

### Analysis of primary outcome measures

The analysis of the coprimary outcomes (ICIQ-UI SF score and IPSS score)^32 33^ will estimate the mean difference (and 95% CIs) between the experimental and comparator arms at 12 weeks postintervention using linear mixed models adjusting for minimisation covariates (reported symptom severity and treatment category), baseline ICIQ-UI SF/IPSS score and other potentially important prognostic covariates. Recruiting centre will be included in the models as a random effect.

### Analysis of secondary outcome measures

Secondary outcomes will be analysed in a similar manner using appropriate generalised linear models. Linear mixed models will be used to analyse Qualiveen and PDQ-8, along with the primary outcomes measures taken at 6 weeks. Bladder diary data will be used to analyse frequency (using oisson regression) and nocturia (using binary logistic regression).

### Interim analyses

No interim analyses are planned.

### Additional analyses

#### Subgroup analysis

Subgroup analyses will be carried out on reported symptom severity and treatment category. Stricter levels of statistical significance (p<0.01) will be sought, reflecting the exploratory nature of these analyses.

#### Economic analysis

The economic analysis will be a within trial evaluation to estimate the cost-effectiveness of TTNS compared with sham TTNS. This evaluation will be conducted from a UK NHS perspective.

The resources required to provide the TTNS treatment will be recorded by the researchers: this will include equipment costs; staff time; training required; and consumables. Participants will complete simple resource use questionnaires at 6 weeks and 12 weeks to record relevant healthcare resource use. Costs will be attached to resource use using appropriate local or national unit cost data, including the Personal and Social Sciences Research Unit^41^ and NHS Reference Costs.^42^ This will demonstrate likely costs of providing TTNS and show key drivers of costs for PwP. Medication will also be recorded at these time points and costs attached using the British National Formulary.^43^

The resource use data will be combined with the coprimary outcome measures in a cost-effectiveness analysis. The results will be presented as a mean incremental cost per unit change in the ICIQ-UI SF and the mean incremental cost per unit change in the IPSS.

### Methods to handle protocol non-adherence and missing data

The analysis will be intention to treat with no account taken of protocol non-adherence. Non-compliance to randomised allocation is not anticipated as participants are blinded throughout.

The extent of missing data will be explored in the outcomes, especially the primary outcomes. Patterns of missing data will be explored and predictors of missingness examined, especially if these vary by intervention. If necessary, multiple imputation will be used to impute missing data assuming the missingness mechanism is missing at random. A detailed statistical analysis plan will be agreed to before the end of data entry and before the treatment code is broken.

### Plans to give access to the full protocol, participant-level data and statistical code

This document is the full protocol. Anyone interested in other data or documentation should contact the corresponding author.

### Oversight and monitoring

#### Composition of the coordinating centre and Trial Steering Committee

The Trial Management Group consisting of the trial manager, admin staff, Chief Investigator and statistician will meet on a biweekly basis to discuss progress and monitor recruitment, data returns and so on.

The TSC will act as the oversight body for the STARTUP trial on behalf of the sponsor and funders. The group will, through regular reports, be responsible for monitoring recruitment and retention rates and patient safety. They will also advise on future continuation of the trial as well as modifying target recruitment or pre analysis follow-up, based on any change to the assumptions underlying the original trial sample size calculation (but not on any emerging differences).

The group will also oversee the timely completion of the final report and appropriate dissemination. A chair with experience of rehabilitation research has been appointed, and the group also has a statistician, Patient and Public Involvement and a clinically experienced person.

### Composition of the data monitoring committee, its role and reporting structure

It was not felt necessary to have a data monitoring committee in this study for a number of reasons: involves non-critical indication; low risk of harm to patients; and treatment is already available.

### Adverse event (AE) reporting and harms

The STARTUP trial involves treatments that are well established in clinical practice; therefore, AEs (although these are unlikely) will be those observed in everyday practice associated with the condition. Expected AEs arising from the treatments are noted below and thus will not be collected as AEs but noted in the weekly follow-up data collection.

#### Urinary tract infection

All AEs and serious AEs will be assessed for seriousness, causality, severity and expectedness and will be reported to the relevant regulatory bodies.

### Frequency and plans for auditing trial conduct

The TSC will meet every 6 months.

### Ethics and dissemination

The study is sponsored by Glasgow Caledonian University, and the START-UP trial office is based in the Nursing, Midwifery and Allied Health Professions Research Unit.

Participants have the right to withdraw from the study at any time and for any reason, and all participants are made aware that withdrawal will not affect their routine care.

## Dissemination

Our dissemination plan is designed to achieve maximum study exposure and impact among a range of beneficiaries (PwP and carers, healthcare professionals, national/local decision makers, professional bodies, patient/carer organisations and the academic research community). Dissemination strategies will include presentations at a range of Parkinson’s disease/dementia/neurology and continence (bladder and bowel) related professional and research conferences. A final report will be submitted to Dunhill Medical Trust and Parkinson’s UK. A summary of the findings will be provided to all study participants (if requested). The main results paper will target a high-impact journal (eg, *BMJ* and *Movement Disorders*), while other papers (eg, methodology and health economics) will target relevant open access journals. Authorship and time scales will be agreed by the project management team. Findings will be provided to NICE and Cochrane Reviews for guideline updates for bladder and bowel dysfunction, diagnosis and management of Parkinson’s disease in primary and secondary care, local guidelines and care pathways and relevant Cochrane Reviews and updates. We will use Twitter to share study news and exploit other relevant social media to raise awareness of study progress and the findings, as well as press releases to news media.

### Plans for communicating important protocol amendments to relevant parties (eg, trial participants and ethical committees)

Funders, sponsors and NHS Research & Development Offices will be notified routinely and appropriate approvals gained and communicated as required by them and by the trial sponsor.

## Discussion

This study is a pragmatic patient-oriented trial aiming to capture a true representation of the actual patient population of interest. We know from previous work and from reviews that there is a lack of evidence-based interventions for bladder dysfunction in PwP. The cost to the NHS and to the patient is considerable, and the effect on QOL both for patients and carers is significant and disabling. The lack of robust evidence on effective management leads to inconsistent advice and confused management pathways. Parkinson’s disease is a long-term condition, and supported self-management is important. TTNS as an adjunct to treatment offers a safe, non-invasive and non-drug intervention that can be undertaken by the patient or a carer at home. As such it is likely to be an attractive option for many. Should the trial demonstrate that TTNS is effective, we feel that an integration into standard local pathways should be possible, as the training required for clinicians and patients (or carers) is minimal.

### Trial status

Subject recruitment is underway. Currently 12 sites are recruiting, and we are ahead of target. The first participant was randomised in October 2018, and recruitment is due to end in June 2020. The TSC has met twice. The trial registration number is https://doi.org/10.1186/ISRCTN12437878.
