# Supplementary material for: Stimulation of the tibial nerve: a protocol for a multicentred randomised controlled trial for urinary problems associated with Parkinson’s disease—STARTUP
Source: BMJ Open. 2020 Feb 17;10(2):e034887. doi: 10.1136/bmjopen-2019-034887 (PMC7044833; doi:10.1136/bmjopen-2019-034887)
Supplement: Supplementary data [file bmjopen-2019-034887supp001.pdf]

STARTUP Research office
NMAHP Research Unit
Glasgow Caledonian University
Glasgow G4 0BA
Tel: 0141 331 3504
Email: Startuptrial@gcu.ac.uk

Trust LOGO, address & contact details

Trial Ref No: 18/ES/0042

Participant ID: ☐☐☐☐☐

Consent Form

A study to asses if transcutaneous tibial nerve stimulation (TTNS) will help with urine leakage in people who have Parkinson's

By signing this form and initialling each box I agree that: **Please initial all boxes**

- I have read and understood the Information Sheet (version X dated XXX) for the above study. I have had the opportunity to consider the information, ask questions and have had them answered satisfactorily. ☐
- My participation is voluntary and that I am free to withdraw at any time, without giving any reason, without my care or legal rights being affected. ☐
- Relevant sections of my medical notes and data collected during the study, may be looked at by individuals from the research office, from regulatory authorities or from the NHS Trust, where it is relevant to my taking part in the STARTUP study. I give permission for these individuals to have access to my records. ☐
- It may not be possible to remove my data from the study once the data analysis has started and results are published. ☐
- I will be randomised and will have a 50% chance of receiving active tibial nerve stimulation and a 50% chance of receiving non-active tibial nerve stimulation ☐
- All relevant data and my contact details can be stored, confidentially and securely by the study offices at Glasgow Caledonian University. ☐

1 for participant; 1 for STARTUP study office; 1 for NHS notes; 1 for study file

Trust LOGO, address & contact details

STARTUP Research office
NMAHP Research Unit
Glasgow Caledonian University
Glasgow G4 0BA
Tel: 0141 331 3504
Email: Startuptrial@gcu.ac.uk

Trial Ref No: 18/ES/0042

Participant ID: | | | | |

By signing this form and initialling each box I agree that:

Please initial all boxes

- I agree to take part in this study ☐
- I agree to my GP being informed of my participation in the study ☐

Your signature (participant) ___________________________ Date_________________

Your name in block capitals ____

To be completed by local STARTUP researcher taking consent

I confirm that I have explained to the person named above, the nature and purpose of the STARTUP trial and the treatments involved

Your signature ____________________________ Date_________________

Your name in block capitals ____

1 for participant; 1 for STARTUP study office; 1 for NHS notes; 1 for study file

McClurg D, *et al. BMJ Open* 2020; 10:e034887. doi: 10.1136/bmjopen-2019-034887
